# Microstructure, hardness and wear behavior of ZrC particle reinforced AZ31 surface composites synthesized via friction stir processing

**DOI:** 10.1038/s41598-023-47381-5

**Published:** 2023-11-16

**Authors:** T. Satish Kumar, Titus Thankachan, S. Shalini, Robert Čep, Kanak Kalita

**Affiliations:** 1 Department of Mechanical Engineering, Amrita School of Engineering, Amrita Vishwa Vidyapeetham, Coimbatore, India; 2 Department of Mechanical Engineering, Karpagam College of Engineering, Coimbatore, India; 3 Department of Physics, PSG Polytechnic College, Coimbatore, Tamil Nadu India; 4 Department of Machining, Assembly and Engineering Metrology, Faculty of Mechanical Engineering, VSB-Technical University of Ostrava, 70800 Ostrava, Czech Republic; 5 Department of Mechanical Engineering, Vel Tech Rangarajan Dr. Sagunthala R&D Institute of Science and Technology, Avadi, Chennai, 600062 India; 6 University Centre for Research & Development, Chandigarh University, Mohali, 140413 India

**Keywords:** Mechanical engineering, Materials science

## Abstract

Dry sliding wear behaviour of friction stir processed (FSP) AZ31 and AZ31/ZrC particles (5, 10, and 15 vol%) reinforced surface composite was investigated at different sliding speeds and loads. The samples were tested using a pin-on-disc apparatus with EN31 steel as the counter body to determine the role of FSP and ZrC reinforcement on the microstructure, hardness, and wear behaviour of AZ31. Base metal AZ31 alloy exhibits a hardness of 60 HV, whereas the 15 vol% ZrC-reinforced composites had the highest hardness of 108 HV. It was also identified that 15 vol% ZrC-reinforced composites exhibited lowest wear rate and friction coefficient under all testing conditions. Abrasion, delamination, oxidation, material softening, and plastic deformation are the primary wear mechanisms viewed from the wear tracks of the samples. Higher volume fraction of ZrC particles exhibited better wear resistance at all speeds and loads than AZ31 alloy. A wear map has been generated for different material compositions and wear conditions to identify the main wear mechanisms easily.

## Introduction

Magnesium and its alloys can be considered as a potential material for automobile, aerospace, biomedical and electronics material owing to its excellent damping capability, machinability, fluidity, corrosion resistance, recyclability etc. The low density of magnesium metal makes it a possible candidate for substitute for replacing conventional metal in automobile industries like steel and aluminum^1–3^. However, comparatively low strength of magnesium hinders its usage in many automobile applications. Plastic deformation in magnesium alloys at room temperature occurs mainly through basal slip while pyramidal and prismatic slips are hindered. Even if prismatic slip happens, it can only offer four independent slip systems along with basal slips which does not comply the von Mises criterion requirements. As the plasticity of magnesium alloys at room temperature is minimal, formability of the same also is minimal which can also be considered as a major factor in automobile industries^4–7^. Studies have proposed that grain refinement can be considered as a major technique in enhancing the strength, ductility and formability of magnesium alloys at room temperature. Several techniques have been developed by the researchers to enhance strength of magnesium and its alloys through severe plastic deformation. However, the ultra-fine grains produced through these techniques leads to generation of high-density dislocations thereby reducing its ductility^3^. Therefore, identifying and developing a processing technique for reducing the grain size of magnesium alloy was the need of the hour making researchers to propose friction stir processing as a possible candidate. Friction stir processing was a thus introduced as a technique to enhance the strength and ductility of magnesium alloys through reducing the grain size of the metal through severe plastic deformation which was then successfully used to for the development of surface and bulk composites^1,2,6,8^.

Friction stir processing, a severe plastic deformation technique developed from the working of friction stir welding process overwhelms the negatives of liquid metallurgy fabrication techniques. During the liquid metallurgy fabrication techniques, processing temperature considered will be above its melting point which will lead to interfacial reactions and other defects. However, when it comes to friction stir processing the processing temperature will be below melting point leading to nil interfacial reactions^9,10^. Composite fabrication through friction stir processing includes (i) compacting of hard ceramic particles into grooves or holes cut into metal substrate, (ii) closing of the groove with the aid of a specifically designed pinless tool and (iii) plunging a rotating friction stir processing tool with pin into the plate and moving it traversal along the plate. During the stirring process, high frictional heat is generated on the substrate metal and this frictional heat along with the vigorous mechanical stirring leads to dynamic recrystallization and homogenous dispersion of ceramic particles.

Several researches have been carried out to study the efficiency of friction stir processing technique in as a grain refinement technique and in dispersing different ceramic particles onto the surface of magnesium alloys^4,9–17^. Jin et al.^18^ friction stir processed AE42 magnesium alloy and observed a reduction in grain size reduction with enhanced mechanical strength and ductility. Jalilvand et al.^9^ proposed friction stir processed AZ31 magnesium alloy and proposed that with increase in the number of traverse passes, grain size reduction occurs radically enhancing the hardness and wear resistance of the surface modified metal. Singh et al.^11^ dispersed titanium carbide onto the surface of pure magnesium through friction stir processing technique and observed a 33% increase in wear resistance. Eivani et al.^7^ carried out friction stir processing of WE43 magnesium alloy and studied the effect of multipasses on its properties. Research proved that with increase in number of traverse passes during friction stir process, grain size of the base metal gets reduced, strength and corrosion resistance increases. Luo et al.^19^ friction stir processed AZ61 alloy at different process parameter to identify the effect of these working conditions on its properties. Vedabouriswaran and Aravindan^20^ dispersed different ceramic particles such as boron carbide, multiwalled carbon nanotubes and a hybrid combination of zirconia and alumina particles onto magnesium RZ5 through friction stir processing technique to study the wear behavior of the developed surface composites. Ram et al.^21^ friction stir processed stir casted magnesium-silicon carbide particles and stated that friction stir processing of as cast magnesium composites leads to porosity eradication, homogenous distribution of reinforcement particles and reinforcement cluster breakage along with grain size refinement. Ti–6Al–4V particles was dispersed onto the surface of AZ31 magnesium alloy employing friction stir processing by Dinaharan et al.^22^ and parametric influence on microstructural evolution and mechanical behavior was studied and analyzed. Hosseinzadeh and Yapici^23^ dispersed silicon carbide onto AZ31 alloy through friction stir processing and stated that addition of silicon carbide enhances the hardness upto 80%. Even though various studies have been made to enhance the characteristics of magnesium alloy through particle dispersion, a minimal literature existed pertaining to the wear behaviour of AZ31/ZrC reinforced surface composites fabricated through FSP. In the current research, an effort has been put forward to investigate the effect of ZrC addition on the microstructure, hardness, and wear behaviour of AZ31 alloy composites.

## Materials and methods

**Figure 1:**
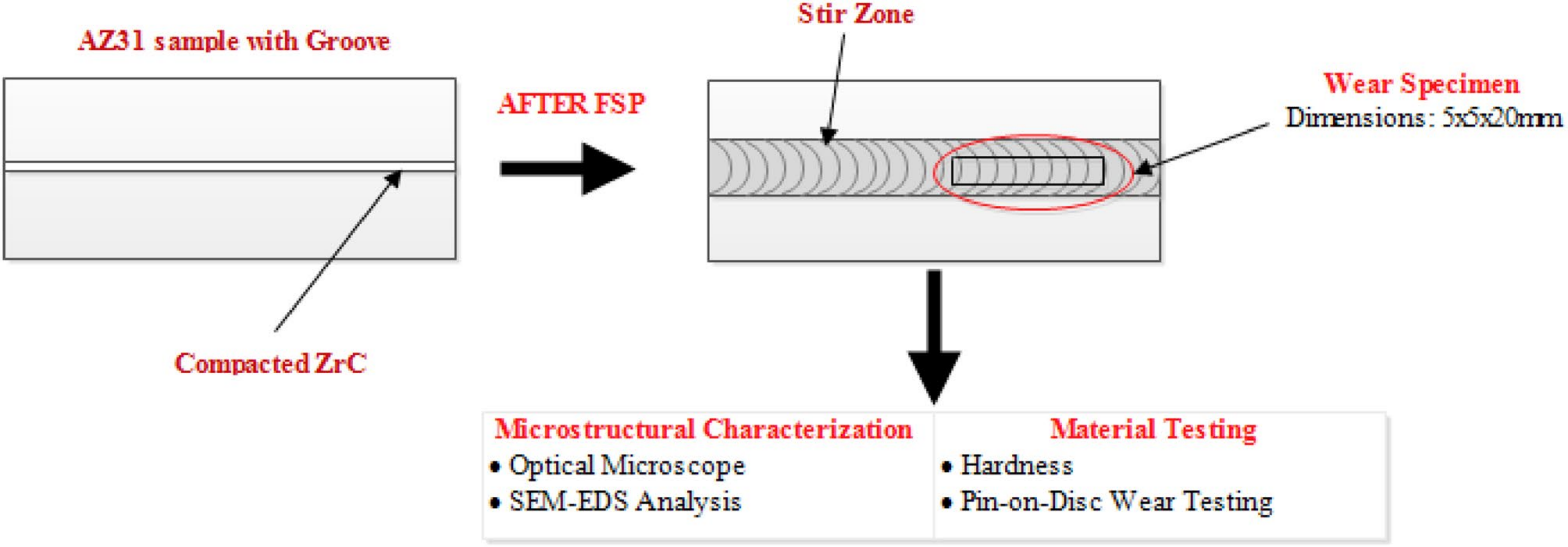
Schematic representation of Mg surface composite fabrication and characterization.

In the current study, ZrC particles with 5–10 µm size and AZ31 alloy were used as reinforcement and matrix materials, respectively. Samples containing 100 × 50 × 10 mm^3^ are machined from AZ31 alloy. Using wire EDM processes a rectangular groove of 4.5 mm depth was cut at the center of the samples; width of the groove was computed using theoretical volume fraction formula. The grooves for compacting ZrC particles are cut at a width of 0.4, 0.9 and 1.3 mm for 5, 10 and 15 volume percentage respectively. The FSP was performed in the current study with a 10 kN axial load, 1200 rpm rotating speed, and 30 mm/min traverse speed. Tool pins with a diameter of 8 mm and 5 mm length were employed to produce the composites. The surface composite samples were polished following ASTM E3-11 procedures before being etched with 70 ml of ethanol, 10 ml of acetic acid, and 4 ml of picric acid solution. XRD analysis was performed using a Shimadzu X-ray diffractometer and Cu K (= 1.5409) radiation. An overall view of the fabrication of the surface composites and its property evaluation details are as shown in Fig. 1.

The worn-out surface was analyzed using a JEOL (JSM-6510LV) scanning electron microscope (SEM) to identify significant wear mechanisms in the produced composites. Using Mitutoyo micro hardness with a 100 g load and a 15-s dwell time, hardness values were measured as indicated by ASTM E 384. The wear test was carried out in a dry sliding condition at room temperature (33 ± 2 °C) using a Ducom-Bangalore pin-on-disc apparatus. The pins of AZ31 alloy and its composites, having dimensions 5.0 × 5.0 × 20.0 mm, was prepared using wire electric discharge machining. The disc is made of EN31 steel, with a hardness of about 65 HRC. The pin sample was polished utilizing various emery sheets up to 1200 grit. The pin samples and steel disc surface were washed with acetone to remove grease or oil. The average of the three samples’ wear values is taken as the final wear rate of the samples. To have a detailed knowledge on the wear behavior of the developed magnesium-based surface composites, wear tests was carried out at different loads (10 N, 40 N, 80 N, 120 N and 160 N), different sliding velocities (1 m/s, 3 m/s, 5 m/s and 7 m/s) for a sliding distance of 2000 m. Wear-tested sample mass loss was measured by means of a weighing balance with 0.1 mg accuracy. The wear-tested sample surface was observed employing SEM to classify the operating wear mechanisms.

## Results and discussion

### XRD examination

**Figure 2:**
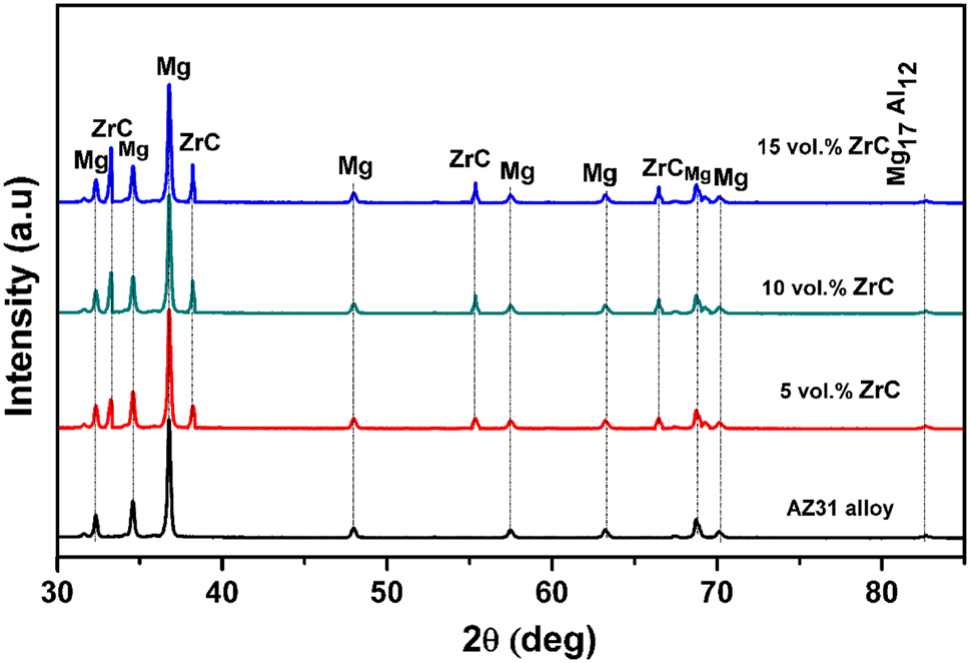
XRD analysis of AZ31/ZrC reinforced composite.

The developed surface composite samples are investigated through the XRD technique to perceive the phases or any compounds that exist in the composites. Figure 2 shows the XRD results of an AZ31 composite with various percentages of ZrC added. The pattern shows the existence of Mg, ZrC, and Mg_17_Al_12_.

### Microstructure analysis

**Figure 3:**
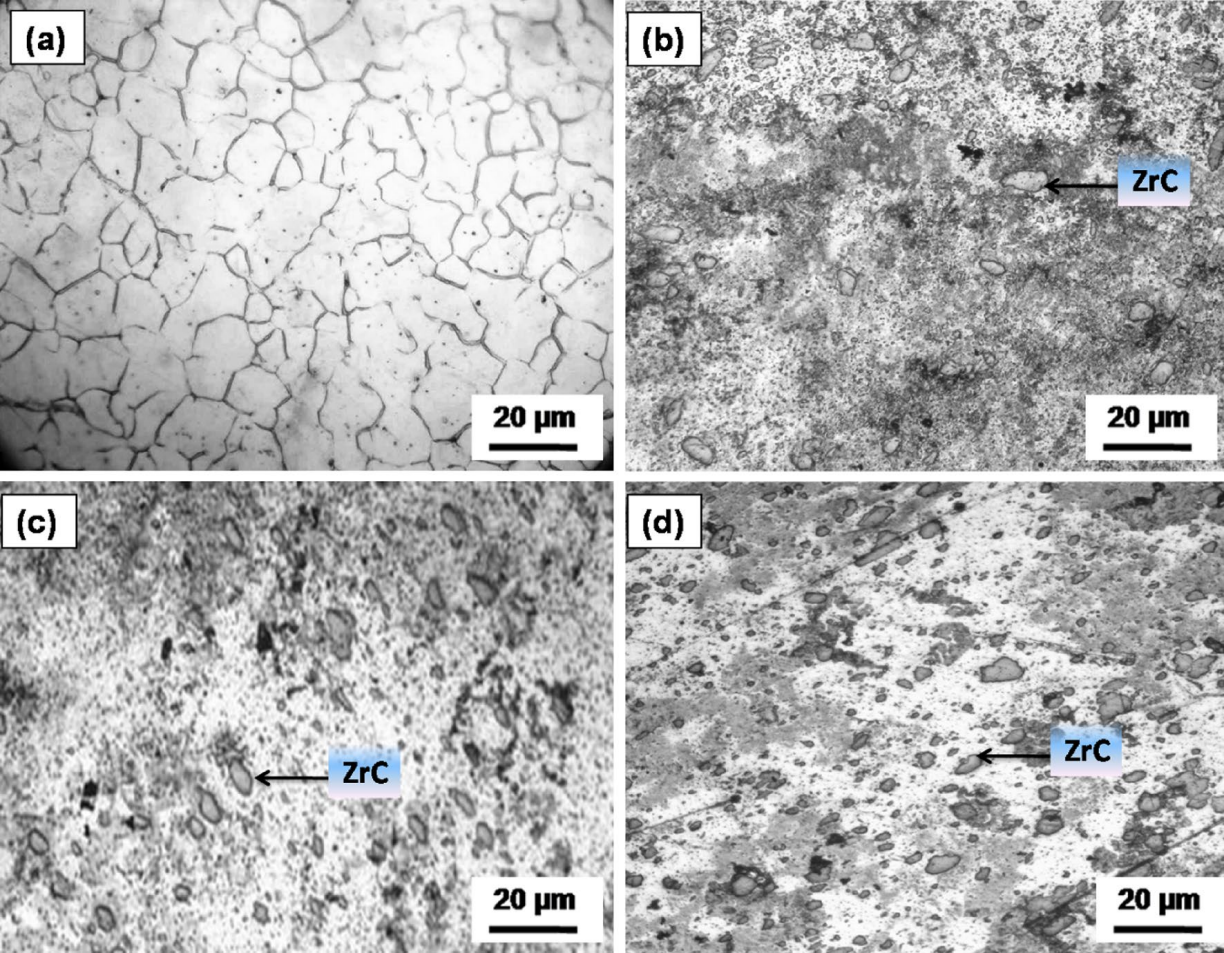
Optical micrograph of (**a**) AZ31 alloy and FSPed composites added with (**b**) 5, (**c**) 10 and (**d**) 15 vol% ZrC.

Microstructural characterization of the developed AZ31 magnesium surface composites dispersed with ZrC particles with the aid of optical microscope is as provided in Fig. 3. It is evident from the microstructures that ZrC particles are distributed throughout AZ31 metal substrate.

The ZrC-particle reinforced composite exhibits significant grain refinement in weld nugget grain size compared to the AZ31 alloy. It is due to the uniform distribution and grain boundary pinning effect of the ZrC reinforcement particles. ZrC particles become obstacles to the growing grain boundary and hinder the further growth of the grain boundary. Weld nugget grain size was found to decrease as the volume percentage addition of ZrC particles increased. The average grain size of the as-received AZ31 alloy is 60 µm, but as the FSP processed samples with a 15 vol% addition of ZrC particles, the matrix grain size was reduced to 5 µm.

**Figure 4:**
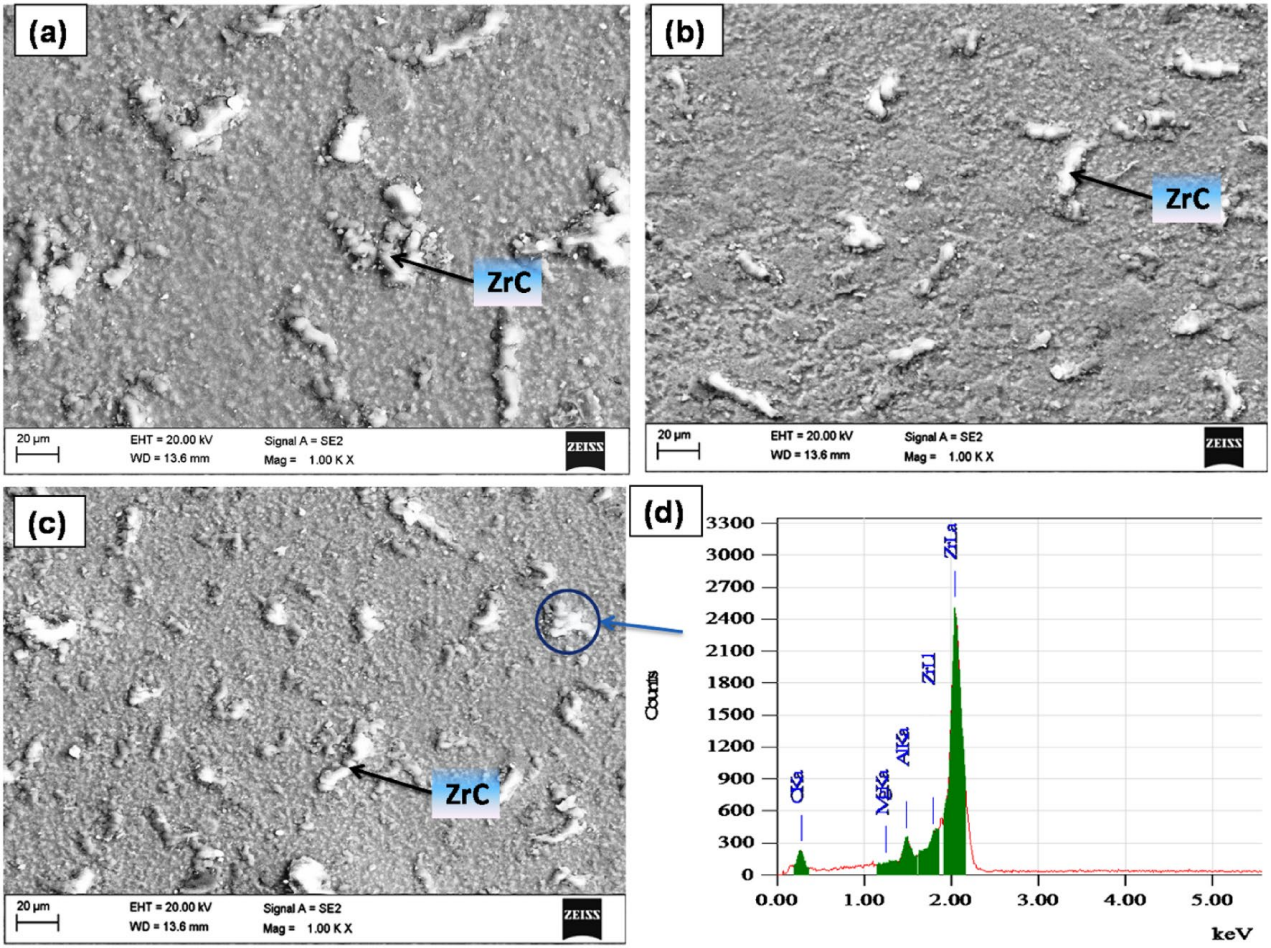
SEM images of AZ31 FSPed with (**a**) 5, (**b**) 10, (**c**) 15 vol% ZrC particles in the stir zone and (**d**) EDS spectra of ZrC added composites.

Figure 4a illustrates the SEM micrographs of the ZrC particles with irregular morphology and a particle size of about 5 µm. Figure 4b–d illustrates the SEM micrographs of the AZ31 alloy reinforced with 5, 10 and 15 vol% ZrC reinforced composites. The SEM image confirms the even distribution of ZrC reinforcement and shows excellent bonding with the AZ31 alloy matrix. With an increase in volume fraction of ZrC particles, the spacing between the particles gets reduced, and it will effectively block the dislocations. To validate the XRD pattern, EDS point analysis has been carried out, as shown in Fig. 4d. The EDS analysis confirms the existence of the added ZrC particles.

### Mechanical properties of AZ31/ZrC surface composites

**Figure 5:**
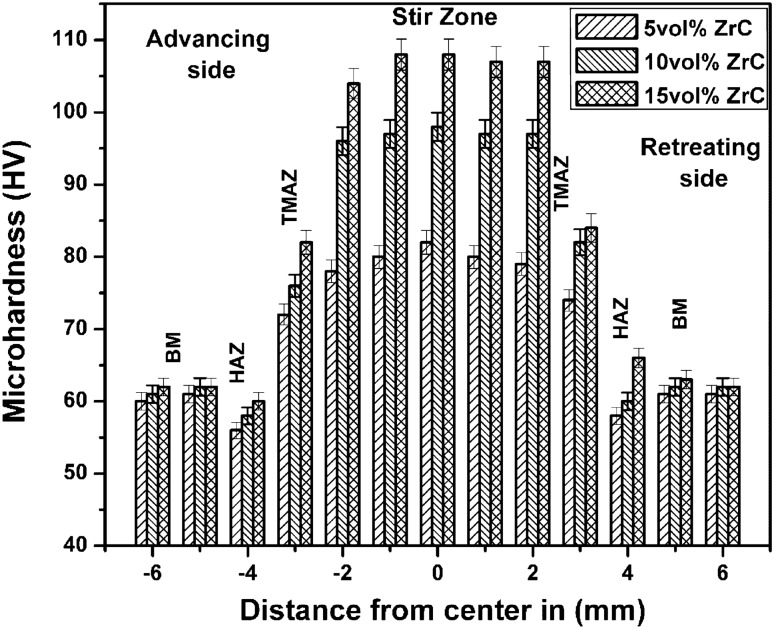
Hardness plot for AZ31/ZrC reinforced composite.

Figure 5 shows the microhardness values that were measured across the FSPed stir zone of the ZrC reinforced AZ31 surface composite. The base AZ31 alloy has a hardness of (60 HV), whereas the FSPed composites were found to exhibit a higher hardness. The hardness increased as the volume percent of ZrC reinforcement increased. In the stir zone, 15 vol% ZrC-reinforced composites had the highest hardness of 108 HV. ZrC ceramic particles will act as heterogeneous nucleation sites, significantly refining the matrix structure. Significant improvement in stir zone hardness was largely due to the following strengthening mechanisms: refined grains, grain boundary strengthening by the Hall–Petch relationship, particle strengthening by the Orowan theory, and the creation of more dislocations owing to the difference in coefficient of thermal expansion between the ZrC reinforcement and the AZ31 alloy matrix^24,25^. The existence of uniformly distributed and fine ZrC particles effectively blocks the dislocation motion^26^. However, the heat-affected region’s hardness was found to be reduced slightly due to the annealing effect and decreased dislocation density.

### Tribological properties of AZ31/ZrC surface composites

**Figure 6:**
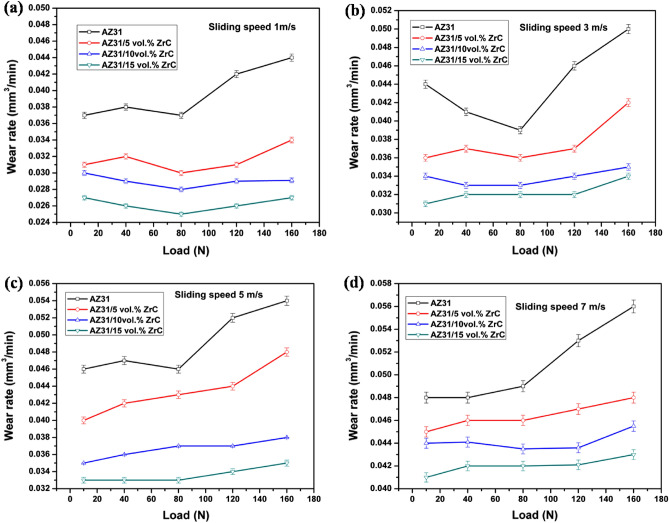
Wear rate of AZ31 alloy and ZrC reinforced composites at various loads and sliding speeds.

Wear rate of the developed magnesium surface composites with respect to increase on load applied and sliding distance is as provided in Fig. 6. It is evident from that figure that with increase in load and sliding distance, wear rate tends to increase. This increase in wear rate with respect to increase in load applied can be attributed to the loosening of dispersed particles at high pressure during the testing process. It can also be observed from Fig. 6a–d that with respect to increase in sliding distance, wear rate tends to increase. This gradual increase in wear rate is due to the melting of specimen pins due to high temperature generated with respect to increase in sliding distance. This leads to removal of protective oxidation layer on the sliding surface of the surface composites. It is also apparent from the Fig. 6 that at all conditions, wear rate of developed surface composites was comparatively higher when compared with the substrate metal. This reduction in wear rate with respect to increase in ZrC addition onto the surface of AZ31 alloy is mainly due to the improved hardness of the developed surface composites. As per Archard’s rule, with increase in hardness of a material wear rate tends to decrease. Influence of applied load on wear rate of AZ31 substrate and surface composites was evaluated by changing the load from 10 to 40 N, 80 N, 120 N and 160 N by keeping sliding distance and velocity constant. Reduction in wear rate with respect to increase in volume fraction of ZrC particles onto the surface of AZ31 Mg alloy is depicted in Fig. 6. As hardness increases, wear resistance of the material tends to increase which is well established as harder materials exhibits better wear and abrasion resistance compared to softer materials. The same is also evident from Fig. 6 that surface composites with high hardness (AZ31 + 15 vol% ZrC) exhibits better wear resistance compared to substrate AZ31 alloy. Along with this, grain refinement and work hardening during FSP can also be put forward as a mechanism that led to increase in wear resistance. ZrC particles being a hard material being dispersed onto the surface of AZ31 magnesium alloy can also be put forward as one of major reason for wear resistance increment with respect particle addition. These uniformly dispersed particles will carry away the load during dry wear testing and also resists the counter material from cutting away the pin sample surface^27^. At low applied load conditions, a negligible wear loss is observed for all the surface composite samples as the contact stress produced due to the loading is made negligible by the composite coating. However as the load increases, the build up stress increases thereby reducing the load carrying compacity of the developed composite coating.

Impact of sliding velocity on the developed surface composites was analyzed at varying sliding velocity of 1 m/s, 3 m/s, 5 m/s and 7 m/s keeping sliding distance and load as constant. It is evident from Fig. 6 that the specimens are dependent on the sliding velocity as with increase in sliding velocity, wear rate of AZ31 and developed surface composites tends to increase. This increase in wear rate can be attributed to high temperature generated at the contact area between the contact pin and hardened disc. This leads to softening of AZ31 matrix metal leading to plastic deformation and henceforth to vigorous wear. Similarly, for surface composites, softening of Mg alloy leads to loosening of ZrC particles leading to wear loss^28^. A dip in wear rate can also be observed in all the samples with respect to increase in sliding velocity as portrayed in Fig. 6a,b which can be due to the presence of oxide layer formed that acts as a protective layer between the contact surface.

**Figure 7:**
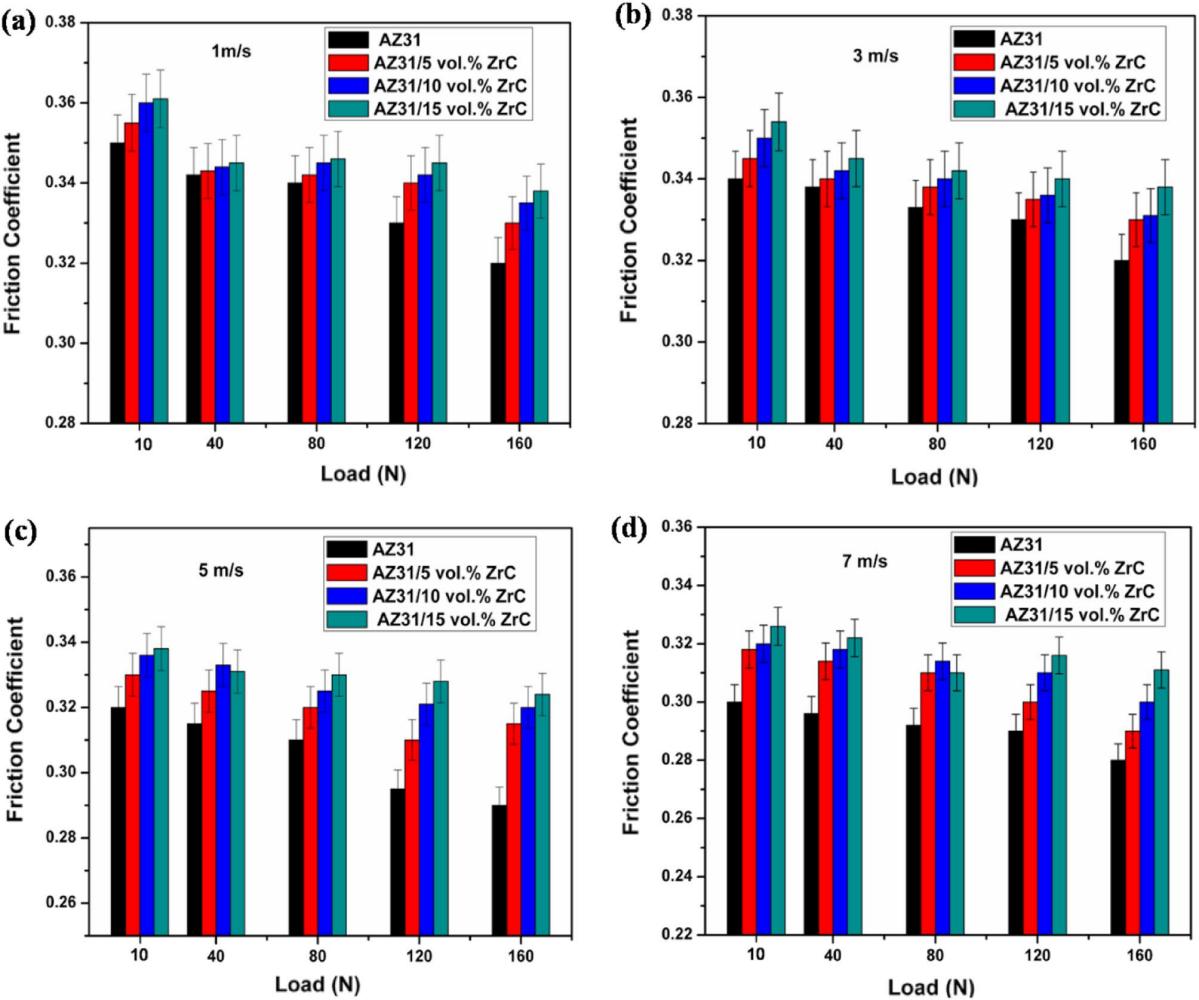
Friction coefficient of AZ31 alloy and ZrC reinforced composites at various loads and sliding speeds.

The frictional coefficient (FC) of specimens with respect to varying load and sliding velocity are as shown in Fig. 7. It is evident from the figure that with increase in ZrC particles into AZ31 alloy, FC tends to increase. This increase in FC can be mainly due to the hard-ceramic particles dispersed onto AZ31 alloy surface which carry away load during wear test. However, when it comes to AZ31 alloy, thermal softening happens due to frictional force reducing FC value. As load and sliding velocity increases, thermal softening happens due to high frictional force which in turn reduces the FC value of the substrate metal (AZ31) and its developed surface composites.

### Wear mechanisms

**Figure 8:**
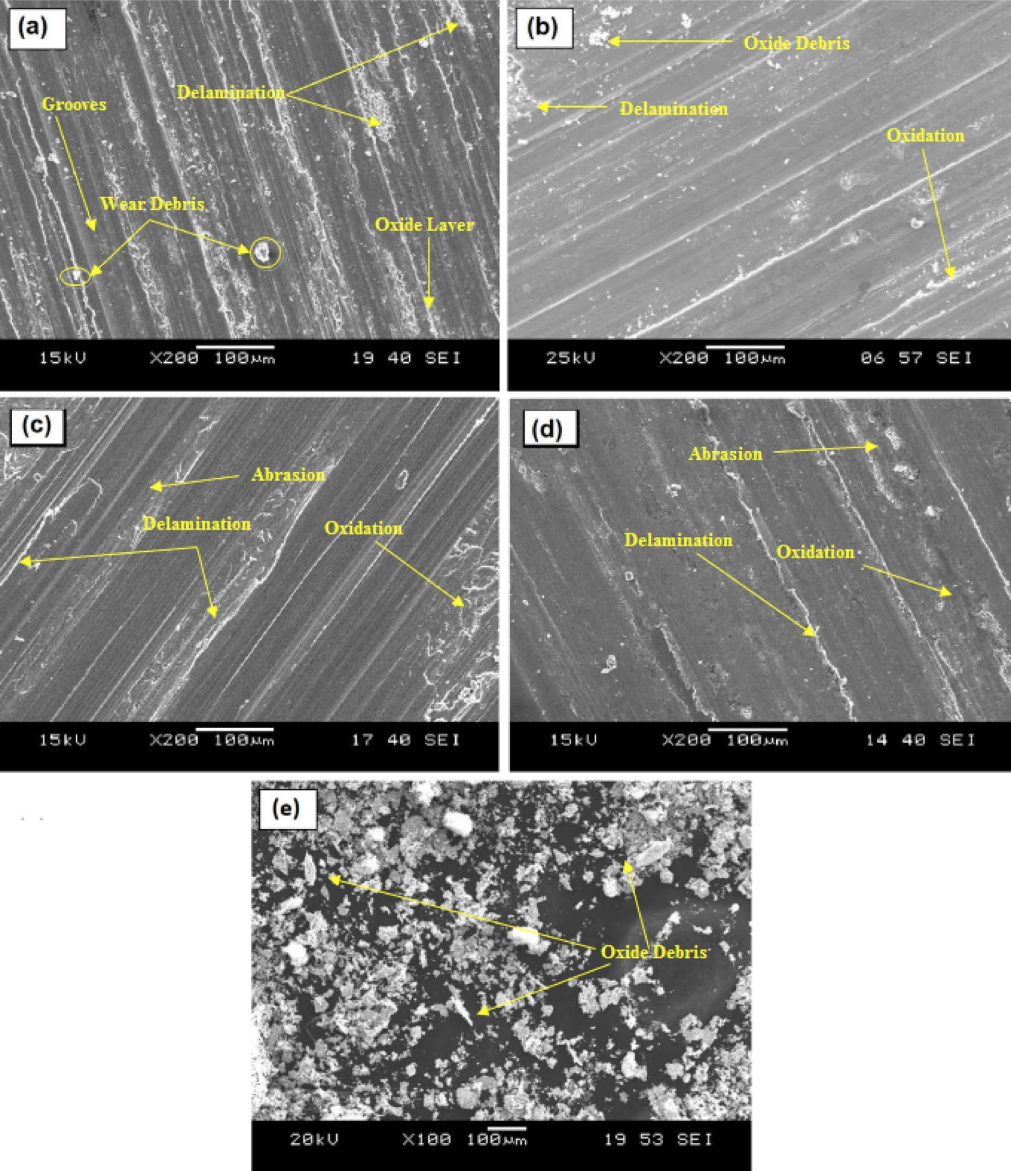
Wear morphology of (**a**) AZ31 at 10 N, (**b**) AZ31 at 80 N, (**c**) AZ31 + 15 vol% ZrC at 10 N, (**d**) AZ31 + 15 vol% ZrC at 80 N and (**e**) wear debris of AZ31 + 15 vol% ZrC.

**Figure 9:**
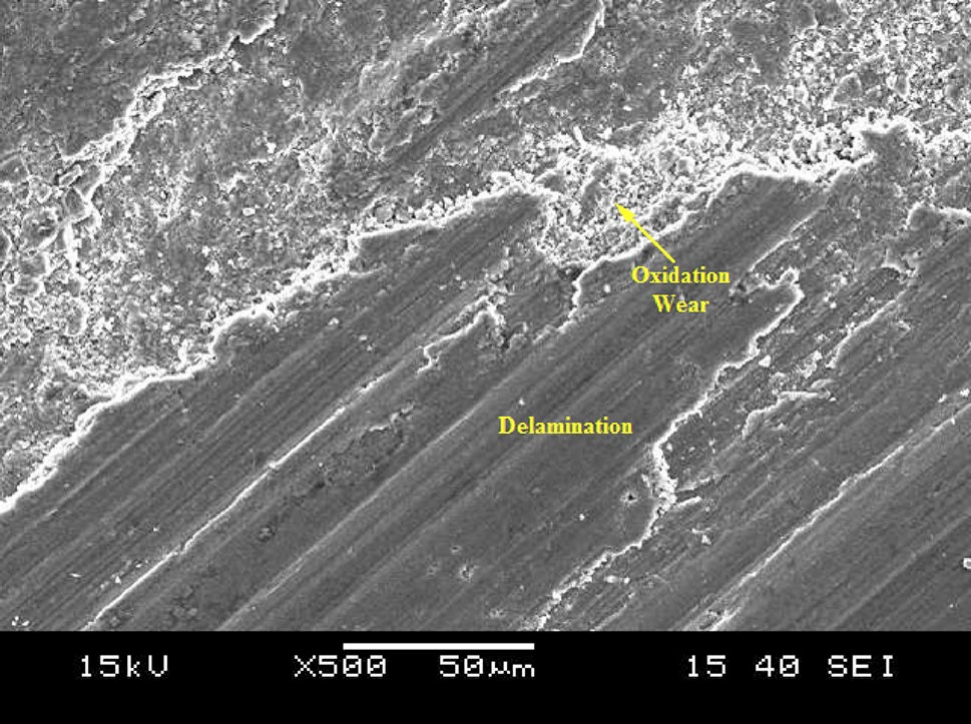
Wear morphology of AZ31 alloy at 160 N.

Wear mechanisms leading to the variation in wear loss and FC value of AZ31 alloy and developed surface composites are analyzed with the aid of SEM images and XRDs. Results portrayed that wear mechanisms include abrasion, oxidation, delamination and melt wear. The worn surface morphology of AZ31 alloy at 10 N and 80 N at a sliding velocity of 1 m/s is as shown in Fig. 8a,b. It is evident that at a load of 10 N, AZ31 alloy exhibits minor grooves over the surface as the dislocation density at the pin surface will be minimum. But with increase in load, dislocation density increases at the pin surface increasing the plastic deformation and henceforth the groove depths. At higher load, the contact area between the pin and disc increases leading to plastic deformation and thereby wear loss to the material. The worn-out surface of AZ31 alloy and its composites revealed the presence of an oxidized layer at all tested sliding speeds and low load conditions. As shown in Fig. 8a, the presence of white spots on a thin layer confirmed the oxidative wear. At high applied loads and sliding speeds, high temperature will be generated between the pin surface and the rotating steel counter disc which results in the melting of the matrix metal. In this case, at high load applied transition of wear mechanism from plastic deformation to melt wear happen as shown in Fig. 9.

Figure 8c,d showcases the worn-out surfaces of AZ31 surface composites dispersed with 15 vol% of ZrC. It can be observed that a mild groove has been formed on the surface of the pin during the wear test at 10 N load as the ZrC particles dispersed on the surface of AZ31 alloy will carry away the load during the analysis. Hardness can also be considered as a major factor in resisting the wear. But as the load increases, pressure at the contact area of the pin increases thereby increasing the contact temperature. This would soften the matrix and loosen the dispersed particles thereby increasing ZrC particle removal from the matrix thereby leading to ploughing out of matrix material which is evident from Fig. 8d.

**Figure 10:**
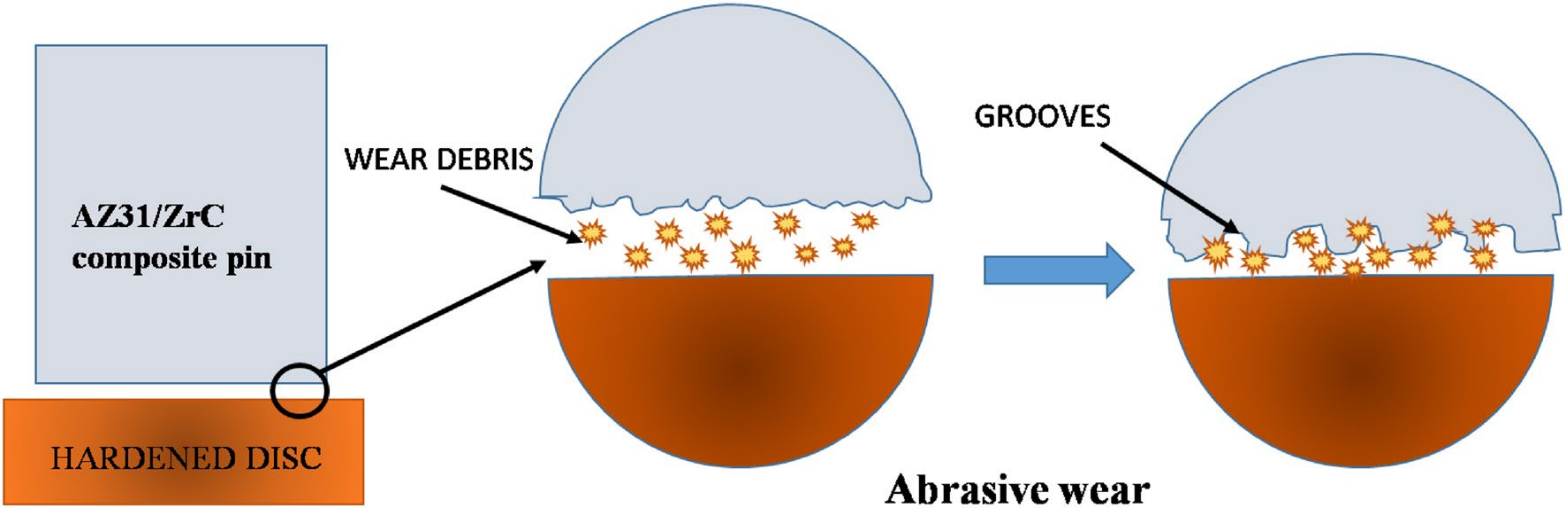
Schematic representation of abrasive wear.

Wear debris of AZ31 surface composites with 15 volume fractions of ZrC particles are shown in Fig. 8e which demonstrates an elongated morphology confirming the ploughing out of material during wear analysis stating the wear mechanism as abrasive mode of wear loss. A schematic representation of abrasive wear is as shown in Fig. 10 which portrays that with increase in load, particles get ploughed out by the hardened counterpart and these wear debris stick on the surface of counterpart and act as a cutting edge to escalate the wear loss of the testing samples.

**Figure 11:**
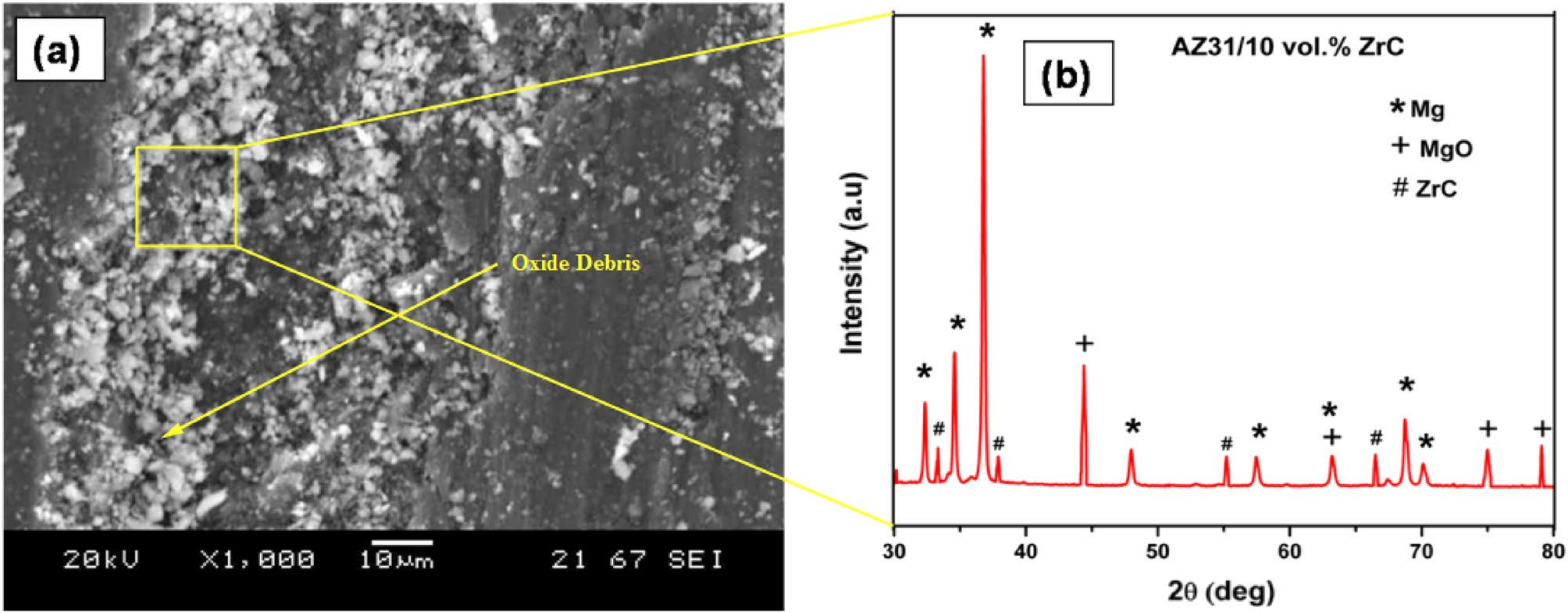
(**a**) Wear debris and (**b**) XRD of AZ31 + 10 vol% ZrC.

SEM and XRD analysis of wear debris proved that the debris was highly oxidized, as shown in Fig. 11a,b, respectively. The frictional heating through sliding leads to oxidation of the pin surface which decreases the wear rate. However, as the oxidized parts get eliminated with further sliding of pin over counter disc leading to wear loss. This oxidized debris will occupy the grooves and voids in the pin surface making a shielding mechanical mixed layer (MML) which in turn reduces the contact between the counter disc and pin thereby reducing wear loss and frictional coefficient of all the samples.

**Figure 12:**
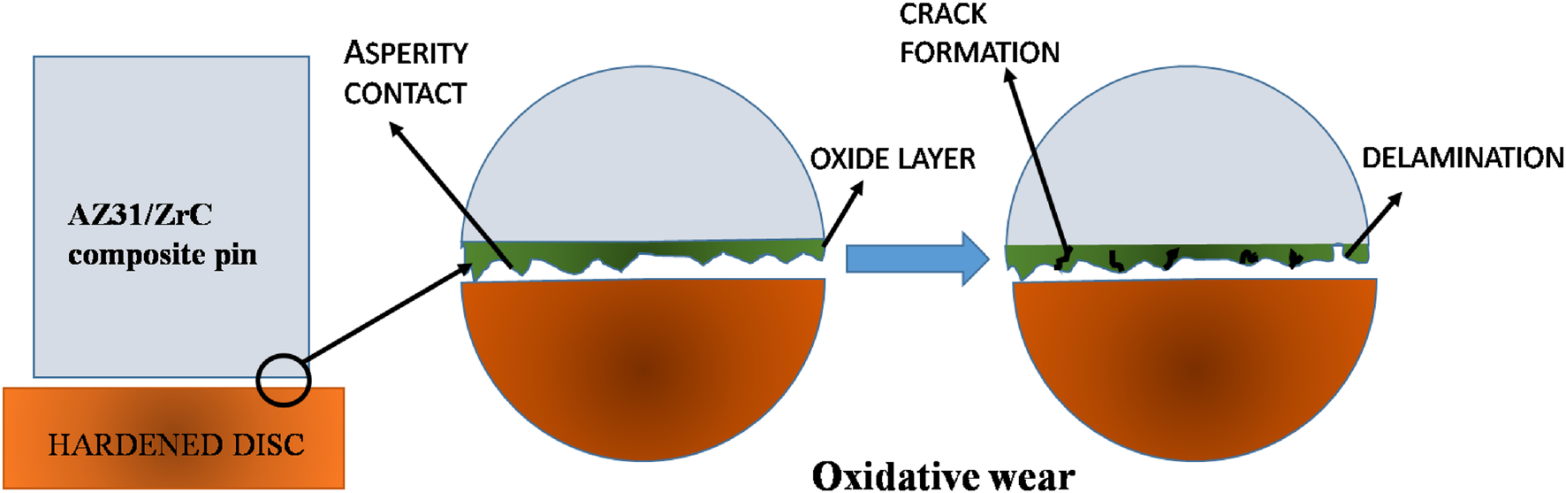
Schematic representation of wear mechanism.

**Figure 13:**
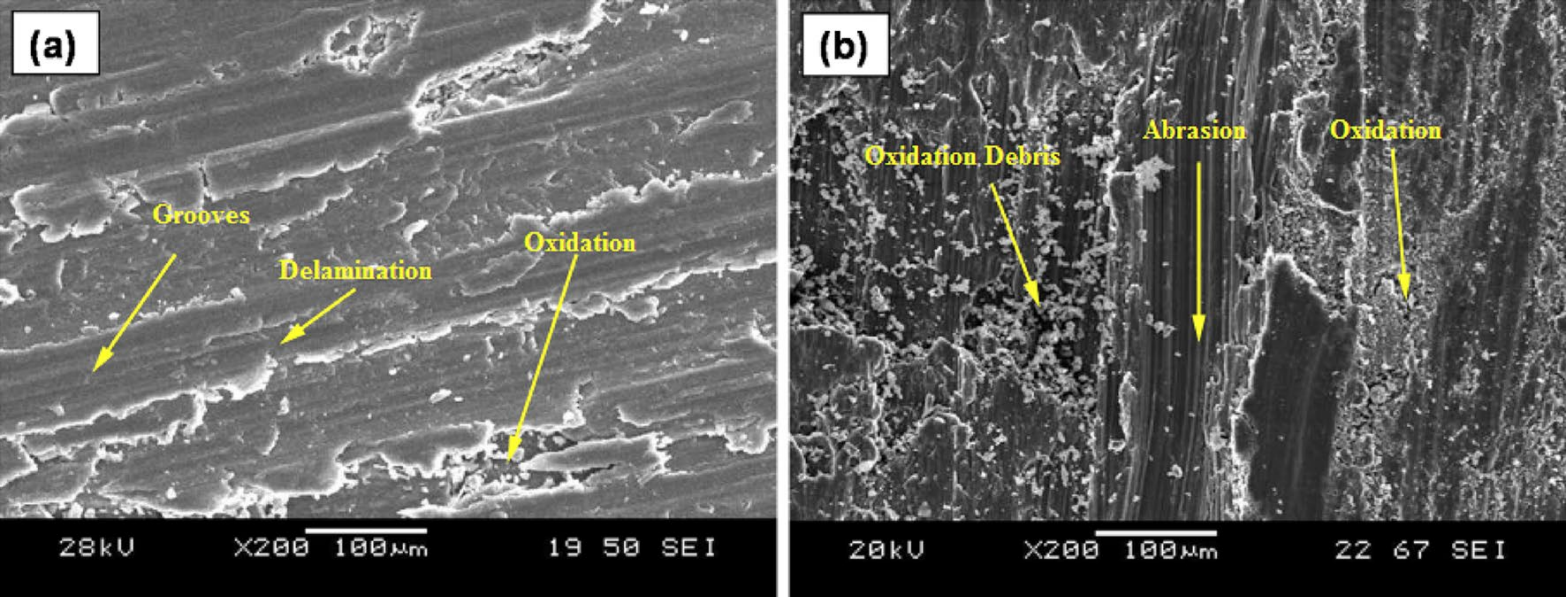
Wear morphology of AZ31 surface composites with (**a**) 10 vol% ZrC and (**b**) 15 vol% ZrC.

A schematic representation of wear that happens during the formation of oxide layer and delamination is shown in Fig. 12. In this research, delamination wear mechanism also happens at higher load conditions, where the worn surface of pin material will be detached in the form of a sheet. Development of cracks and subsurface cracks along the sliding direction leads to the detachment of pin materials. Representative delamination wear morphology of AZ31 surface composites with 10 vol% ZrC and 15 vol% ZrC tried at 5 m/s, and 40 N is shown in Fig. 13. The presence of a large number of sheet-shaped debris is confirmed by SEM images of wear debris (Fig. 13a,b). Continuous sliding causes fatigue and lead to surface deformation, crack formation, and its propagation.

Consequently, it was also evident from this research that delamination wear increases with increase in load. Composites are primarily prone to delamination wear due to crack formation and propagation at the ZrC-AZ31 matrix interface. Composites exhibited a low wear rate when tested at 3 m/s due to the formation of an oxide layer. When sliding speed is above 3 m/s a rise in wear rate is observed; propose that the oxidative wear mechanism change to a delamination wear mechanism. Comparatively the developed surface composites exhibit better resistance as the composites reinforced with ZrC particles exhibited superior resistance to plastic deformation.

**Figure 14:**
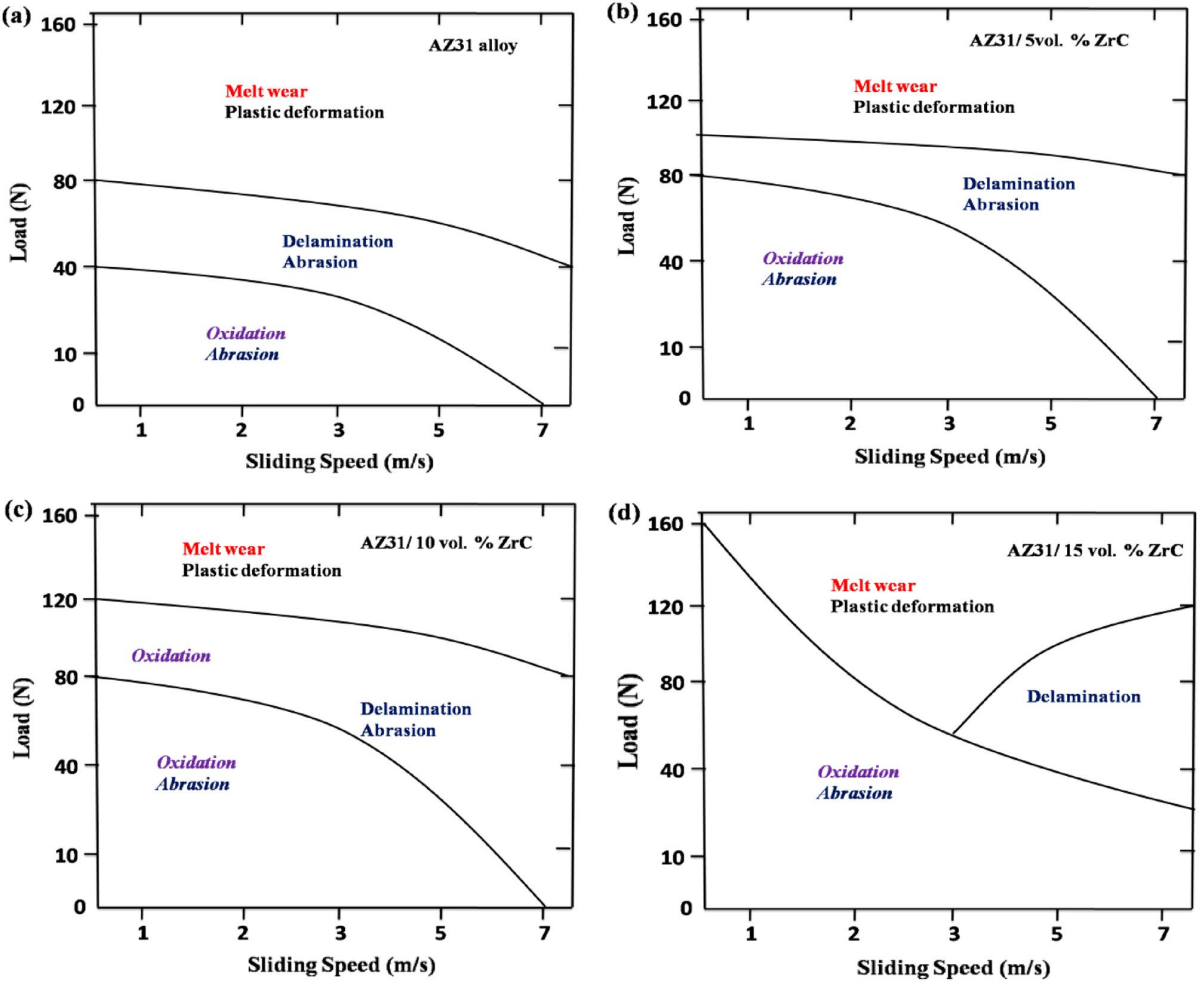
Wear mechanism maps of (**a**) AZ31alloy, (**b**) AZ31/5 vol% ZrC, (**c**) AZ31/10 vol% and (**d**) AZ31/15 vol% ZrC.

Investigation of worn tracks of the wear-tested surface, wear rate, and friction coefficient allows for the creation of wear maps with sliding speed and applied load conditions, as shown in Fig. 14. In Fig. 14, each zone describes the dominant wear mechanism, and lines separate their transition. The wear mechanisms of AZ31 alloy and AZ31/ZrC composites are as follows: Irrespective of the sliding speed, at low loads, oxidation and abrasion are the primary wear mechanisms for both AZ31 alloy and AZ31/ZrC composites. For the AZ31 alloy, at higher loads, the wear mechanisms changes from abrasion and oxidation to delamination due to subsurface cracks thereby increasing the wear rate. This transition in the wear mechanism for AZ31 alloy takes place above 40 N. AZ31 alloy exhibited an increase in wear rate at high speeds and loads, and the wear mechanism changed from delamination to melt wear. In contrast, the ZrC reinforced composites requires higher loads and speeds for the wear transition to delamination wear. For AZ31/5 vol% ZrC-reinforced composites with slow speed, the transition occurs above 80 N, and for AZ31/10 vol% ZrC-reinforced composites, the transition happens below 80N. Temperatures above 120N and speeds of 5 m/s are required for transition in ZrC composites of 100 N and 15 vol% ZrC-reinforced composites. This behavior can be attributed to (i) the formation of a protective oxide layer^29–34^ over the surface reduces the wear rate and delays the transition of the wear mechanism and (ii) increase in hardness of the composites with increase in ZrC addition. Higher ZrC particle volume percent promotes the formation of a more compact oxide layer, which protects the pin surface at low speeds and up to 120 N of load. In AZ31 surface composites, the presence of ZrC particles delays the plastic deformation and, in turn, reduces the wear rate.

## Conclusions

AZ31 alloy exhibited various wear mechanisms concerning load and speed; at low loads (10–40 N), oxidation is the dominant wear mechanism; at middle loads (40–80 N), delamination and abrasion are dominant wear mechanisms; and at high loads (greater than 80 N), melt wear plays a major role. In contrast, oxidative wear is predominantly observed in composites, which reduces the composites wear rate by forming a protective oxide layer. Melt wear is the most common wear mechanism observed in AZ31 alloy and AZ31/ZrC reinforced composites at higher loads (120–160 N) and sliding speeds (5–7 m/s). The addition of ZrC reinforcement particles appreciably improved the wear resistance of AZ31 alloy by delaying the change in wear mechanism due to the development of a more stable oxide layer with an increase in vol% of ZrC particles, and it also provided resistance to plastic deformation of the matrix alloy.

## Data Availability

The data presented in this study are available through email upon request to the corresponding author.
